# The Use of Artificial Intelligence for Estimating Trabecular–Iris Angle from Slit-Lamp Images Developed Using Anterior-Segment Optical Coherence Tomography

**DOI:** 10.3390/bioengineering13101156

**Published:** 2026-10-03

**Authors:** Takahiro Mizukami, Jun Ohashi, Hiroki Nishimura, Ryota Yokoiwa, Yuji Takayanagi, Hirotsugu Soga, Shintato Nakayama, Eisuke Shimizu

**Affiliations:** 1 Yokohama Keiai Eye Clinic, Yokohama 2400065, Japan; hiroki@ouiinc.jp (H.N.); y.takayanagi1008@gmail.com (Y.T.); h.sogaa@gmail.com (H.S.); ophthalmolog1st.acek39@keio.jp (E.S.); 2 OUI Inc., Tokyo 1070062, Japan; junohashi.work@gmail.com (J.O.); ryota.yokoiwa@gmail.com (R.Y.); p.shintaro@ouiinc.jp (S.N.); 3 Department of Ophthalmology, Keio University School of Medicine, Tokyo 1608582, Japan

**Keywords:** glaucoma, ResNet50, smart eye camera, trabecular iris angle

## Abstract

**Background:** Narrowing of the trabecular–iris angle (TIA) is a key anatomical feature of primary angle-closure disease and contributes to impaired aqueous humor outflow; however, conventional angle assessment requires specialized expertise or costly imaging equipment. This study aimed to develop an artificial intelligence model for quantitative TIA estimation from anterior-segment images acquired with a smartphone-attached slit-lamp microscope. **Methods:** In this retrospective study, 127,512 candidate frames were extracted from anterior-segment videos obtained from 2654 eyes. After automated diagnosability filtering, 59,412 frames suitable for TIA500 estimation were retained and assigned to the training, validation, and test datasets using patient-level grouping. A ResNet50-based model was trained to estimate inferior, nasal, and temporal TIA500 values using the corresponding measurements obtained by anterior-segment optical coherence tomography (AS-OCT) as the ground-truth reference. Model performance was evaluated by comparing the AI-predicted values with the AS-OCT measurements using mean absolute error (MAE), root mean squared error (RMSE), Spearman’s rank correlation coefficient, and the intraclass correlation coefficient (ICC). **Results:** Per-eye MAEs were 7.01° ± 5.69°, 7.28° ± 5.80°, and 7.90° ± 6.37° for inferior, nasal, and temporal TIA, respectively. Corresponding correlations with reference measurements were 0.683, 0.696, and 0.646, while ICCs were 0.769, 0.764, and 0.743 (all *p* < 0.001). **Conclusions:** These findings demonstrate the feasibility of estimating TIA500 from smartphone-based slit-lamp images. However, the moderate correlations with AS-OCT measurements indicate that the present model is not yet sufficiently accurate for independent clinical diagnosis or screening, and further model improvement and external validation are required.

## 1. Introduction

Primary angle-closure glaucoma (PACG) is a cause of blindness, characterized by anatomical narrowing of the anterior chamber angle, which obstructs aqueous humor outflow and elevates intraocular pressure [1,2]. Together with its earlier stages, primary angle-closure suspect (PACS) and primary angle closure (PAC), PACG constitutes the spectrum of primary angle-closure disease (PACD) [1]. The global burden of PACD is significant; a recent meta-analysis estimated that approximately 17.1 million people aged over 40 years were affected by PACG globally in 2020, and this number is projected to surpass 23 million by 2040 due to aging populations [1]. The disease disproportionately affects East Asian and other Asian populations, who account for approximately 70% of all cases, and its prevalence is notably higher among older adults and females [3]. One of the key anatomical risk factors includes a narrow trabecular–iris angle (TIA); specifically, eyes with crowded anterior segments characterized by shallow anterior chamber depth (ACD), thicker crystalline lenses, or an anteriorly positioned lens have reduced angular width between the iris and trabecular meshwork, predisposing them to angle closure [3,4]. In PACS, an occludable narrow angle, defined by at least 180° of iridotrabecular contact observed on gonioscopy, is present without elevation of intraocular pressure or evidence of optic nerve damage [5]. Progression to PAC may occur when pupillary block mechanisms induce anterior bowing of the peripheral iris, leading to apposition against the trabecular meshwork, particularly during mid-dilation. This mechanism can be further exacerbated by anatomical features such as plateau iris configuration [3].

Traditional methods for detecting narrow angles and angle-closure disease have significant limitations in accessibility and scalability. Gonioscopy—direct visualization of the anterior chamber angle using a goniolens—is considered the clinical gold standard for diagnosing angle closure [5]. However, this method is inherently subjective, requiring specialized skills and proper technique, which results in notoriously low adoption rates in general clinical practice and large-scale screening programs. Consequently, many cases of angle closure remain undiagnosed, particularly in resource-limited settings where clinicians trained in gonioscopy are insufficient to effectively screen large populations [5]. Simpler proxy methods, such as the Van Herick technique—which estimates peripheral anterior chamber depth using slit-lamp examination—are available but have significant limitations. For instance, one study reported that approximately 78% of eyes classified as “open” by the Van Herick technique were found to have occludable angles upon gonioscopic examination [6]. This highlights that relying on coarse estimation methods can lead to missing a significant proportion of eyes at risk of angle closure [5]. An alternative method, anterior-segment optical coherence tomography (AS-OCT), provides high-resolution, cross-sectional imaging of the anterior chamber angle, along with quantitative metrics such as TIA, angle-opening distance, and trabecular–iris space area [7]. AS-OCT can objectively detect narrow angles that may be clinically overlooked and demonstrates a high negative predictive value, making it an effective screening tool capable of identifying at-risk eyes even in the absence of an expert examiner [7]. However, AS-OCT devices are costly and non-portable, making them inaccessible for many developing regions where angle-closure disease is highly prevalent [8]. Indeed, in numerous high-risk communities, the use of OCT-based imaging is limited by prohibitive costs and infrastructure constraints [8].

In recent years, artificial intelligence (AI), particularly deep learning, has emerged as a highly effective tool in medical image analysis, including ophthalmology [9]. Notably, AI-driven diagnostic systems have achieved remarkable success predominantly in retinal disorders compared to anterior segment diseases [10]. High-profile accomplishments, such as automated screening for diabetic retinopathy, detection of age-related macular degeneration, and assessment of glaucomatous optic neuropathy based on fundus photography, illustrate that deep learning systems can match or even surpass expert ophthalmologists in retinal image interpretation [11]. To date, most ophthalmic AI research and commercial applications have concentrated on posterior segment disorders, particularly retinal diseases and glaucoma, while anterior segment conditions have recently begun to attract focused investigation [12,13]. Several factors may explain the lag in anterior-segment AI research: (1) retinal diseases depend primarily on single-modality imaging, whereas anterior-segment disorders require multiple examination types [11]; (2) challenges in standardizing diverse anterior-segment image data [14]; and (3) absence of universally accepted gold-standard diagnostic criteria [15]. However, global research hotspots have recently shifted toward anterior segment diseases, particularly cataracts and keratopathies [16]. To address these challenges and in line with current trends, we utilized a smartphone-based slit-lamp microscope to develop a deep learning model capable of estimating anterior chamber depth. This was achieved by training the model on AS-OCT-derived parameters obtained from smartphone images, thereby facilitating the collection of large volumes of standardized data [17,18].

In this study, we propose a novel solution: a deep-learning model capable of estimating the TIA from standard slit-lamp videos. We trained the model on a paired dataset of slit-lamp and AS-OCT images, using the AS-OCT-derived TIA measurements as ground-truth labels.

## 2. Materials and Methods

### 2.1. Ethics Approval Statement

This study adhered to the tenets of the Declaration of Helsinki and was approved by the Institutional Ethics Review Board of Minamiaoyama Eye Clinic, Tokyo, Japan (Approved No #202101; 20 July 2021). Given the retrospective design and use of deidentified data, the requirement for written informed consent was waived with the approval of the Institutional Ethics Review Board.

### 2.2. Study Design

In this retrospective study, we collected all slit-lamp videos recorded at Yokohama Keiai Eye Clinic (Kanagawa, Japan) between July 2020 and March 2023 using a smartphone-attached slit-lamp microscope (Smart Eye Camera, SEC; SLM-i07/SLM-i08SE, OUI Inc., Tokyo, Japan) [17]. Videos were acquired with direct focal illumination, a 0.1~0.3 mm full-height slit beam angled at 40°, and were required to show both the cornea and crystalline lens in focus for at least five seconds. Inclusion criteria were (1) eyes with clear visualization of the cornea; and (2) a minimum of five seconds of video duration. We excluded videos with severe corneal opacities (e.g., bullous keratopathy) and inadequate cornea visualization. After exclusions, 2654 eyes remained (Figure 1). Corresponding TIA measurements were obtained by AS-OCT (CASIA2 Advance, Tomey, Nagoya, Japan) under standardized lighting. The SEC and AS-OCT examinations were performed during the same clinical session under the same standardized ambient illumination conditions. Participants maintained primary gaze during both examinations. For SEC imaging, each participant was instructed to look straight ahead, and anterior-segment videos were acquired using a fixed imaging geometry, with the slit beam angled at 40°. Pharmacologic mydriatic or miotic agents were not administered before either examination. Pupil diameter was not quantitatively measured.

In this study, the videos were first decomposed into still frames, yielding 127,512 candidate frames from 2654 eyes. At this stage, the extracted frames included images unsuitable for TIA estimation because of blinking, partial or complete eyelid closure, motion blur, defocus, or inadequate visualization of the relevant anterior-segment structures. We therefore applied our previously developed diagnosability extraction algorithm to automatically identify frames suitable for subsequent analysis [18]. After this filtering process, 68,100 unsuitable frames were excluded, leaving 59,412 diagnostically acceptable frames for model development and evaluation.

To prevent information leakage, dataset partitioning was performed with patient-level grouping. Patients were assigned to the training, validation, and test datasets so that the number of eyes was distributed in an approximate ratio of 6:2:2. When both eyes of the same patient were included, both eyes were assigned to the same dataset. Consequently, all videos obtained from a given patient and all frames extracted from those videos were retained exclusively within a single dataset. No patient, eye, video, or frame was shared among the training, validation, and test datasets. The final datasets comprised 1641 eyes and 36,829 frames for training, 529 eyes and 11,881 frames for validation, and 484 eyes and 10,702 frames for testing. The network was trained to predict TIA500 values using AS-OCT-derived measurements as the ground-truth reference, and its performance was evaluated using only the independent test dataset.

### 2.3. Smartphone-Attached Slit-Lamp Microscope

We used the SEC, a smartphone-attached slit-lamp microscope, to record anterior-segment videos. Previous studies have shown that its diagnostic performance is comparable to that of conventional slit-lamp microscopes in both animal models and clinical settings [17]. To compile a large dataset of anterior-segment images, we recorded videos using iPhone 7 and iPhone SE 2 devices (Apple Inc., Cupertino, CA, USA) at resolutions of 720 × 1280 to 1080 × 1920 pixels and frame rates of 30 or 60 fps.

### 2.4. Trabecular–Iris Angle Measurement Using Anterior Segment Optical Coherence Tomography

AS-OCT imaging was performed using the CASIA2 Advance system during the same clinical session as SEC imaging and under the same standardized ambient illumination conditions. Participants maintained primary gaze, and no pharmacologic mydriatic or miotic agents were administered. Certified optometrists acquired at least two high-quality, pupil-centered scans per eye using the manufacturer’s standard anterior-segment protocol [17]. TIA500 measurements were obtained at 270° (inferior), 0° (nasal), and 180° (temporal). TIA500 was used as the representative angle parameter in this study, as it has been reported to demonstrate high reproducibility in anterior chamber angle measurements [19,20]. TIA500 is defined as the angle formed with its apex at the iris recess, with one arm passing through a point on the trabecular meshwork located 500 μm from the scleral spur and the other arm intersecting the iris perpendicularly opposite to this point [21]. For each eye and anatomical direction, the TIA500 values obtained from at least two acceptable AS-OCT scans were averaged and used as the ground-truth reference. Scans affected by poor centration, motion artifacts, inadequate image quality, or unreliable automated delineation were excluded, and imaging was repeated until at least two acceptable scans were obtained. All scans and automated segmentation outputs were independently reviewed by a certified optometrist (H.N.) and an ophthalmologist (E.S.). Any disagreement regarding image acceptability was resolved by discussion and consensus. Automated segmentation and scleral-spur localization were not manually modified; scans with unacceptable automated delineation were excluded and reacquired.

### 2.5. Datasets and Machine Learning

All videos were downloaded from the cloud server to a local workstation and decomposed into individual frames. A previously developed “Diagnosable extraction algorithm” was then applied to select frames clearly showing the cornea, anterior chamber, and crystalline lens for TIA estimation (Accuracy of 0.994) [18,22]. After the general diagnosability filtering and patient-level dataset partitioning described in Section 2.2, frame eligibility was assessed separately for each anatomical direction. Frames unsuitable for estimating TIA500 in a particular direction were excluded from the analysis for that direction. Consequently, the direction-specific datasets contained different numbers of frames and partially overlapped because a single frame could contribute to more than one directional analysis. Each eligible frame was paired with the corresponding AS-OCT-derived TIA500 value at 270° (inferior), 0° (nasal), or 180° (temporal).

Three separate ResNet50-based regression models were trained independently to estimate TIA500 at 0° (nasal), 180° (temporal), and 270° (inferior), respectively [23]. Each model used frames eligible for analysis of its corresponding anatomical direction and the paired AS-OCT-derived TIA500 measurements as the ground-truth reference. The models were trained from scratch using randomly initialized weights, without pretrained weights or transfer learning. Input images were resized to 224 × 224 pixels and normalized using the standard ImageNet channel-wise statistics (RGB means: 0.485, 0.456, and 0.406; RGB standard deviations: 0.229, 0.224, and 0.225). No data augmentation was applied. For each model, the original classification layer of ResNet50 was replaced with a fully connected regression layer with a single linear output corresponding to TIA500 in the relevant anatomical direction. The model was trained using a masked mean squared error loss, so that only available direction-specific ground-truth labels contributed to the loss calculation. Stochastic gradient descent was used as the optimizer, with a learning rate of 0.001, a batch size of 8, and a prespecified total of 50 epochs. Training and validation losses were recorded throughout the 50 epochs to assess model convergence and potential overfitting. Validation MAE and RMSE were also calculated at each epoch for each anatomical direction. No early-stopping procedure was applied. For each anatomical direction, the checkpoint with the lowest validation loss was selected for evaluation on the independent test dataset.

The global step counted the cumulative number of training mini-batches processed. Each epoch comprised 3199, 3203, and 937 training mini-batches for the nasal (0°), temporal (180°), and inferior (270°) models, respectively. For visualization, epoch boundaries were identified using the global steps recorded at the end-of-epoch validation events. Training loss was calculated as the arithmetic mean of the logged training-loss values within each epoch, and validation metrics were recorded at the end of each epoch. All learning curves were plotted against epoch over 50 epochs. The total loss consisted solely of the TIA regression loss with a weight of 1. Therefore, the two losses were identical, and only one loss series was displayed for each dataset. Validation MAE and RMSE were expressed in degrees.

For eye-level evaluation, predictions from eligible frames belonging to the same eye were combined using the median for each anatomical direction. Grad-CAM heatmaps were generated as described in Section 2.6 [24]. Model training and inference were performed on a workstation equipped with an Intel^®^ Core™ i9-13900H CPU and an NVIDIA^®^ GeForce RTX™ 4090 GPU.

### 2.6. Statistical Analysis

As this represents an exploratory investigation, formal sample-size calculations were not feasible; instead, all eligible cases were included to maximize study power. Model prediction accuracy was evaluated using mean absolute error (MAE) and root mean squared error (rMSE) calculated per-eye (aggregated across all frames) and per-frame at the inferior (270°), nasal (0°), and temporal (180°) angles with standard deviation (SD) calculations. Spearman’s rank correlation coefficient assessed agreement between AI-predicted and AS-OCT-measured values. We calculated the intraclass correlation coefficient (ICC) to assess reliability between AI-predicted and ground-truth measurements, using a two-way mixed-effects model for absolute agreement (ICC [3,K]), with individual scores reflecting the degree of measurement concordance. All analyses were conducted in SPSS version 29 (IBM Corp., Armonk, NY, USA).

To visualize class activation mapping, we employed Grad-CAM, a state-of-the-art post hoc interpretability technique [24]. Grad-CAM highlights regions of interest that contribute to a deep neural network’s prediction by computing the gradients of the target class score concerning the input image and projecting them onto the final convolutional feature maps, as in previous studies [18,25].

## 3. Results

### 3.1. Diversity of the Data Set

A total of 127,512 candidate frames were extracted from anterior-segment videos obtained from 2654 eyes. After automated diagnosability filtering, 68,100 frames unsuitable for TIA500 estimation were excluded, leaving 59,412 diagnostically acceptable frames for model development and evaluation. Of these, 36,829 frames from 1641 eyes were included in the training dataset, 11,881 frames from 529 eyes in the validation dataset, and 10,702 frames from 484 eyes in the test dataset. Among the 59,412 diagnostically acceptable frames, the numbers of frames eligible for analysis differed by anatomical direction because some frames did not adequately visualize the structures required to estimate TIA500 in a particular direction. Accordingly, 41,662 frames from 1986 eyes were analyzed at 0°, 41,704 frames from 1972 eyes at 180°, and 16,508 frames from 608 eyes at 270°. Because the direction-specific datasets overlapped, with some frames and eyes contributing to more than one direction, these counts are not mutually exclusive and their sum exceeds the total number of unique frames and eyes. Figure 2 shows the distribution of TIA500 measured with the CASIA2 at 270°, 0°, and 180° in the study population.

### 3.2. Model Training and Convergence

The learning curves for the three independently trained models are shown in Figure 3. For all anatomical directions, training loss decreased markedly during the initial epochs and continued to decrease more gradually thereafter. Validation loss, MAE, and RMSE generally decreased during the early training period and became relatively stable during the later epochs, with some fluctuations. No sustained deterioration in validation performance was observed during the later training period. However, training loss remained lower than validation loss, and the stabilization of validation metrics alone does not exclude overfitting.

### 3.3. Model Prediction Accuracy with Ground Truth

The overall per-eye TIA 270° predictions yielded an MAE of 7.01° ± 5.69° and an rMSE of 9.01° ± 12.60°, whereas per-frame predictions showed an MAE of 7.76° ± 6.21° and an rMSE of 9.94° ± 13.36°. For the TIA 0°, per-eye predictions achieved an MAE of 7.28° ± 5.80° and an rMSE of 9.30° ± 11.19°, compared with per-frame values of 7.79° ± 6.11° and 9.90° ± 11.87°, respectively. At TIA 180°, per-eye performance was an MAE of 7.90° ± 6.37° and an rMSE of 10.14° ± 12.57°, versus per-frame MAE of 8.32° ± 6.71° and rMSE of 10.68° ± 13.41°. In all results, aggregate (per-eye) predictions outperformed per-frame estimates in both MAE and rMSE.

### 3.4. Correlation Between the Model and Ground Truth

Per-eye predictions demonstrated strong correlations with AS-OCT measurements: at TIA 270° (*r* = 0.683; 95% CI, 0.563–0.774; *p* < 0.001), TIA 0° (*r* = 0.696; 95% CI, 0.637–0.747; *p* < 0.001), and TIA 180° (*r* = 0.646; 95% CI, 0.580–0.704; *p* < 0.001). Per-frame predictions also correlated strongly but less so: at TIA 270° (*r* = 0.627; 95% CI, 0.601–0.652; *p* < 0.001), TIA 0° (*r* = 0.646; 95% CI, 0.632–0.659; *p* < 0.001), and TIA 180° (*r* = 0.607; 95% CI, 0.592–0.622; *p* < 0.001). In all results, aggregate per-eye estimates outperformed pre-frame predictions.

### 3.5. Agreement Between the Model and Ground Truth

When aggregating frames per eye, the model demonstrated good reliability with ICCs of 0.769 (95% CI, 0.659–0.844; *p* < 0.001) at TIA 270°, 0.764 (95% CI, 0.710–0.808; *p* < 0.001) at TIA 0°, and 0.743 (95% CI, 0.685–0.791; *p* < 0.001) at TIA 180°. Per-frame analysis yielded slightly lower but still strong ICCs of 0.737 (95% CI, 0.714–0.758; *p* < 0.001) at TIA 270°, 0.739 (95% CI, 0.727–0.751; *p* < 0.001) at TIA 0°, and 0.719 (95% CI, 0.705–0.732; *p* < 0.001) at TIA 180°. In all cases, aggregate per-eye estimates outperformed individual-frame predictions.

### 3.6. Representative Examples of AI Angle Prediction with Heatmap Visualization

Three representative images of TIA at 270°, 0°, and 180° are presented, each overlaid with a color-coded activation heatmap generated by Grad-CAM, where warmer colors indicate regions of higher model attention (Figure 4). The Grad-CAM visualizations suggested that the model primarily emphasized regions around the iridocorneal angle when estimating TIA500, although some attention was also distributed across adjacent anterior-segment structures.

## 4. Discussion

This study presents a novel approach for quantitatively estimating the TIA from smartphone-captured slit-lamp images using AS-OCT-derived TIA500 measurements as the ground-truth reference. We developed a paired dataset comprising slit-lamp videos and corresponding AS-OCT measurements from 2654 eyes and trained a ResNet50-based deep-learning model to estimate TIA500 at 270° (inferior), 0° (nasal), and 180° (temporal). In the independent test dataset of 484 eyes, the per-eye mean absolute errors were 7.01 ± 5.69°, 7.28 ± 5.80°, and 7.90 ± 6.37°, respectively. The corresponding Spearman correlation coefficients were 0.683, 0.696, and 0.646, while the ICCs were 0.769, 0.764, and 0.743, respectively. Thus, the predicted values showed moderate correlations and good agreement with the AS-OCT measurements. Per-eye estimates consistently outperformed individual-frame predictions, suggesting that aggregating predictions across multiple video frames may improve measurement reliability. These findings support the feasibility of quantitative TIA estimation using a portable smartphone-based slit-lamp system. Nevertheless, given the moderate correlations and observed prediction errors, the current model should be regarded as a proof-of-concept rather than a substitute for gonioscopy or AS-OCT, particularly because prediction errors may affect the classification of eyes near clinically relevant narrow-angle thresholds. Further model improvement and external validation across different populations, institutions, and imaging devices are required before its utility in clinical decision-making or population screening can be established.

Previous research in a Caucasian population reported a mean temporal TIA500 of 27.04° ± 12.04° and a mean nasal TIA500 of 27.43° ± 11.75° [26]. In the current dataset, both the temporal and nasal TIA500 values were higher, which may be due to anatomical differences linked to racial variation. Additionally, the distribution of TIA500 values in our cohort showed no significant skew, indicating a balanced representation within the sampled population. The model we developed in this study demonstrated a MAE of 7.01 ± 5.69° and an rMSE of 9.01 ± 12.60° in an independent test set comprising 2654 eyes. This error range is comparable to the measurement reproducibility of AS-OCT itself and falls within the clinically acceptable margin. Quantitative estimation of TIA is valuable for risk assessment in clinical practice (e.g., TIA < 20° indicating high risk), and the majority of prediction errors were within a range unlikely to result in clinically significant misclassification [27]. Grad-CAM was included as an exploratory, qualitative tool to provide a general indication of the image regions contributing to TIA500 estimation. However, Grad-CAM does not establish that the highlighted regions have a causal role in the model’s predictions and cannot independently explain the magnitude or cause of prediction errors. A systematic analysis of high-error cases using clinical image review and additional interpretability methods will be necessary in future studies to better characterize potential failure mechanisms.

We previously developed an AI algorithm capable of estimating ACD using the SEC, a smartphone-attached slit-lamp microscope [17]. We demonstrated that its accuracy was comparable to that of external datasets using similar slit-lamp images and AS-OCT images [22]. The conventional Van Herick method has limited sensitivity and specificity for detecting angle closure, particularly in cases with moderate TIA, where false negatives and false positives are common [28]. In contrast, our proposed model predicts TIA as a continuous variable, offering more nuanced and clinically useful information than binary classification. A related study reported that AS-OCT achieved a median sensitivity of 87% and a median specificity of 84% for detecting angle closure, demonstrating high diagnostic performance across various clinical settings [5]. Our model achieves comparable accuracy using simpler slit-lamp photographs, highlighting its potential for accessible and reliable angle assessment. The heatmap overlays further demonstrate that the AI focuses on the correct anatomical region—specifically, a bright signal at the angle structure—when making its assessment, thereby enhancing the interpretability of the algorithm’s decisions [17]. These illustrative cases demonstrate the system’s potential in a clinical setting: the AI can take a standard slit-lamp photograph, highlight the angle area, and output a quantitative TIA that closely matches an OCT measurement, thereby facilitating automated angle assessment in ophthalmic practice.

By utilizing the smartphone-based slit-lamp device known as the SEC, we were able to acquire high-quality anterior segment images without the need for conventional, expensive, and stationary equipment [17]. Furthermore, extracting frames from recorded videos enabled the inclusion of diverse imaging conditions, thereby enhancing the variability and robustness of the training dataset. To improve prediction accuracy, we implemented a two-stage AI architecture consisting of high-quality image selection followed by TIA estimation, ensuring that only diagnostically reliable images were used for model training [18]. Collectively, these innovations contributed to the development of a model with superior generalizability and precision compared to conventional approaches. A key strength of the current model is its ability to quantitatively measure the TIA—a task traditionally done with AS-OCT imaging and trained specialists—using a more affordable, portable imaging system combined with AI technology. This innovation offers potential for many applications, including glaucoma risk screening in remote or underserved areas, basic eye exams in temporary clinics or shelters during natural disasters, anterior segment screening in general medical or primary care settings, and remote image analysis in teleophthalmology. Since PACG remains a leading cause of blindness in Asia, early detection and preventive treatments, such as laser peripheral iridotomy, are vital for preserving vision [29,30]. Although no studies have directly evaluated the combined use of AI-based ACD estimation and TIA measurement in the same eye, previous research shows that each parameter can be accurately obtained using AI [17,31]. Evidence also suggests that models integrating multiple anterior segment parameters outperform those relying on a single measure [15]. Therefore, combining AI-derived ACD and TIA, which capture complementary aspects of anterior segment anatomy, may enhance the sensitivity and specificity of screening for PAC and PACG. Consequently, the widespread adoption of these tools could significantly benefit public health, especially in regions with limited access to specialized eye care.

Deep learning has been applied to heterogeneous medical imaging modalities for a wide range of detection and classification tasks. Iqbal et al. used transfer learning for the automated detection of knee-joint synovial fluid on magnetic resonance images, demonstrating the potential value of adapting pretrained representations to specialized medical imaging tasks with limited domain-specific data [32]. Another study applied a deep convolutional neural network to the automated multi-class classification of dermoscopic skin lesions and emphasized the importance of evaluating model performance across heterogeneous pathological appearances using multiple diagnostic metrics [33]. In contrast to these detection and classification tasks, the present study used a regression model to estimate TIA500 as a continuous anatomical parameter from smartphone-captured slit-lamp images. Although pretrained weights were not used in the present model, transfer learning may represent a useful approach for improving model efficiency and generalizability in future studies. External validation across different populations, institutions, and imaging devices will also be required.

This study has several limitations. First, the model focuses only on the TIA as a structural parameter and does not include other indicators of glaucoma severity, such as visual field defects, intraocular pressure, or optic nerve status; therefore, it cannot determine the presence or progression of glaucoma. Second, all data were collected from Japanese subjects, and given known racial differences in ocular anatomy—such as corneal shape, axial length, and angle configuration—the applicability of the model to other populations remains unverified. Third, image acquisition was limited to a single type of device (the Smart Eye Camera; SEC), and further studies are needed to test compatibility with other slit-lamp systems and smartphone models. A previous study using the SEC showed that image enhancement altered interobserver reliability in the assessment of anterior-segment findings: appropriate enhancement improved the evaluation of tear break-up time and corneal fluorescein staining, whereas excessive enhancement reduced reliability for some ocular-surface features [34]. These findings suggest that image-processing parameters may also affect the visibility of anterior chamber structures and the performance of TIA estimation. Future studies should therefore evaluate whether task-specific image enhancement can improve TIA prediction while avoiding the loss or distortion of relevant anatomical information. Fourth, although the SEC and AS-OCT examinations were performed during the same clinical session under the same standardized ambient illumination conditions and without pharmacologic dilation or miosis, pupil diameter was not quantitatively measured. Small physiologic differences in pupil size between examinations may therefore have affected the anterior-chamber angle. In addition, pixel-level spatial registration between the SEC and AS-OCT images was not performed because the two modalities use different imaging geometries. Correspondence was established at the eye and anatomical-direction levels, which may have introduced variability into the ground-truth labeling process. Fifth, although no skew was observed in the distribution of TIA500 values in the current dataset, selection bias may still be present because the data were collected in a clinical rather than a population-based setting. Additionally, since TIA500 values may vary among different racial groups, further validation is needed in populations with diverse ethnic backgrounds [26]. Sixth, the present Grad-CAM analysis was limited to representative cases and was not designed as a systematic failure analysis of images with the largest prediction errors. Future studies should examine high-error cases in relation to image quality, acquisition conditions, ocular anatomy, and model attention patterns. Seventh, the hierarchical dependence among multiple frames obtained from the same eye and between both eyes of the same patient was not fully addressed in the present statistical analysis. Future studies should employ patient- or eye-level resampling methods and include Bland–Altman agreement analysis, threshold-based diagnostic-performance metrics, and angle-range-specific error analyses to more comprehensively evaluate the model’s screening performance. To address these limitations, future external validation involving multiple institutions, ethnic groups, and imaging devices is essential. Additionally, comprehensive AI models that include functional and multimodal indicators—such as visual fields, intraocular pressure, and optic nerve evaluation—should be developed.

In ophthalmic screening, a certain degree of measurement error may be acceptable; however, minimizing false negatives—i.e., avoiding missed diagnoses—is of paramount importance. With a mean absolute error of approximately 7°, our model demonstrates sufficient accuracy for practical use, particularly in risk-based screening scenarios employing thresholds such as TIA < 20° [27]. Furthermore, the methodology proposed in this study can be extended to other anterior segment parameters, such as ACD or lens vault, thereby contributing to the broader advancement of AI-based anterior segment analysis. As such, this work represents an important first step toward enabling AI-driven ophthalmic diagnostics in resource-limited settings where AS-OCT is unavailable.

## 5. Conclusions

We developed an AI-based model that quantitatively estimates inferior, nasal, and temporal TIA from smartphone-captured slit-lamp images, achieving mean absolute errors of approximately 7–8° and good agreement with AS-OCT measurements. These findings demonstrate the feasibility of using a portable imaging system for AI-assisted assessment of anterior chamber angle anatomy. However, the model is not intended to replace gonioscopy or AS-OCT and cannot independently diagnose angle-closure disease or glaucoma. External validation across diverse populations, institutions, and imaging devices, together with evaluation of clinically relevant screening thresholds, is required before clinical implementation. If validated, this approach may support more accessible angle-closure risk assessment, particularly in community-based and resource-limited settings.

## 6. Patents

OUI Inc. has the patent for the Smart Eye Camera and relations (Patent No. JP; 6627071, USA; 12193745, EU; 19743494.7, China; 201980010174-7, India; 541687, VN; 1-2020-04893, and Africa; AP6569, AP6839. Patent pending JP; 2019-198354, 2021-535472, 2022-500489, 2024-205339. EU; 20846667-2, 2175926.2, US; 17/799043, VN; 1-2022-01003, Africa; AP/P/2022/013851). There are no other relevant declarations relating to these patents.

## Figures and Tables

**Figure 1:**
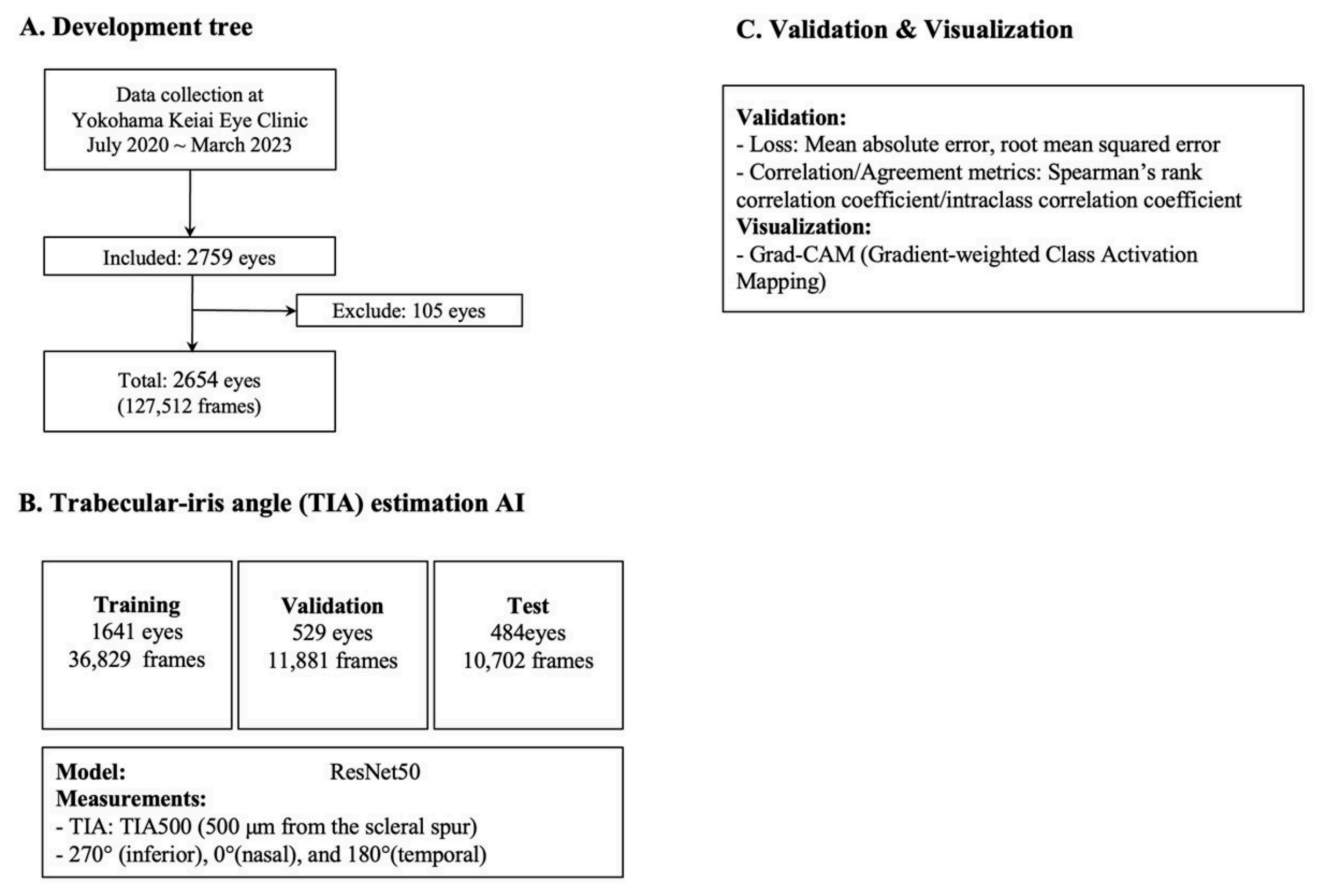
Study flow and development of the artificial intelligence (AI) model for trabecular–iris angle (TIA) estimation. (**A**) Anterior-segment videos were collected from 2759 eyes at Yokohama Keiai Eye Clinic between July 2020 and March 2023. After excluding 105 eyes, 127,512 candidate frames were extracted from videos obtained from 2654 eyes. Automated diagnosability filtering excluded 68,100 frames affected by blinking, eyelid closure, motion blur, defocus, or inadequate visualization of the relevant anterior-segment structures. The remaining 59,412 diagnostically acceptable frames were included in model development and evaluation. (**B**) Diagnosable frames were allocated to the training, validation, and test datasets. A ResNet50-based model was trained to estimate anterior-segment optical coherence tomography–derived TIA500 values at 270° (inferior), 0° (nasal), and 180° (temporal). (**C**) Model performance was evaluated using mean absolute error, root mean squared error, Spearman’s rank correlation coefficient, and intraclass correlation coefficient. Gradient-weighted class activation mapping (Grad-CAM) was used to visualize image regions contributing to the model’s predictions.

**Figure 2:**
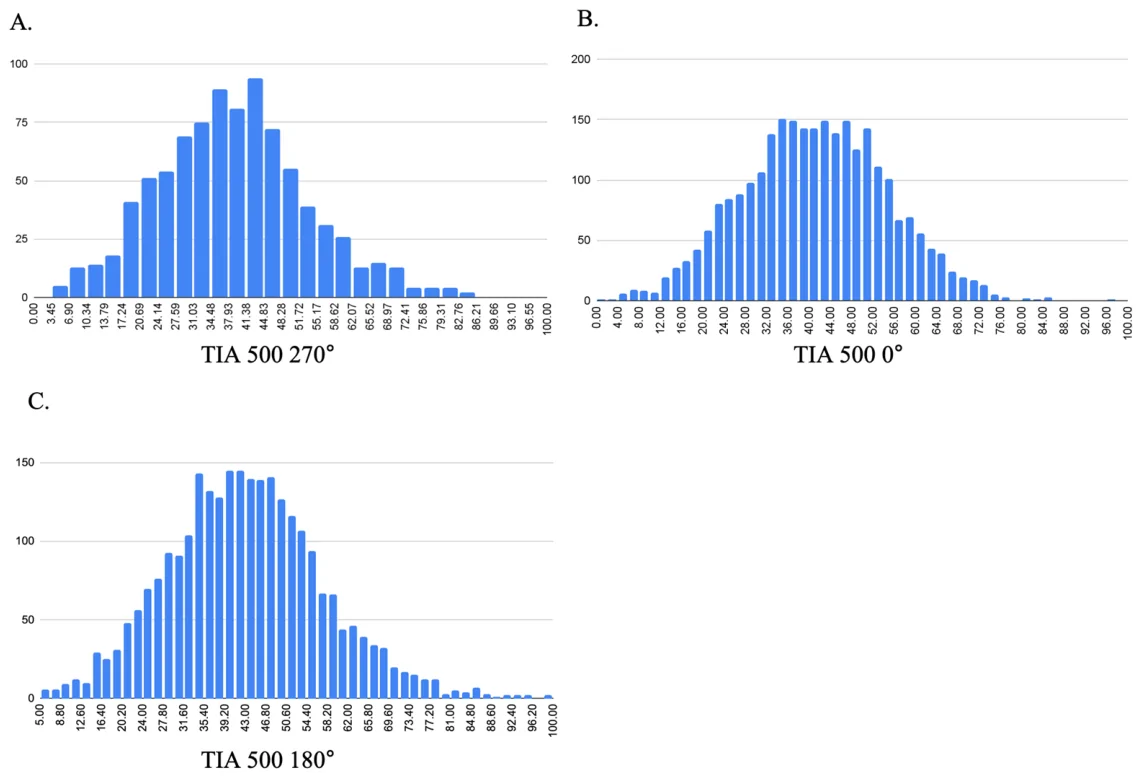
Distribution of TIA measurements in the study population. Histograms show the distributions of TIA500 values measured using anterior-segment optical coherence tomography (CASIA2) at (**A**) 270° (inferior), (**B**) 0° (nasal), and (**C**) 180° (temporal). The horizontal axis represents TIA500 in degrees, and the vertical axis represents the number of eyes. TIA500, trabecular–iris angle measured 500 μm from the scleral spur.

**Figure 3:**
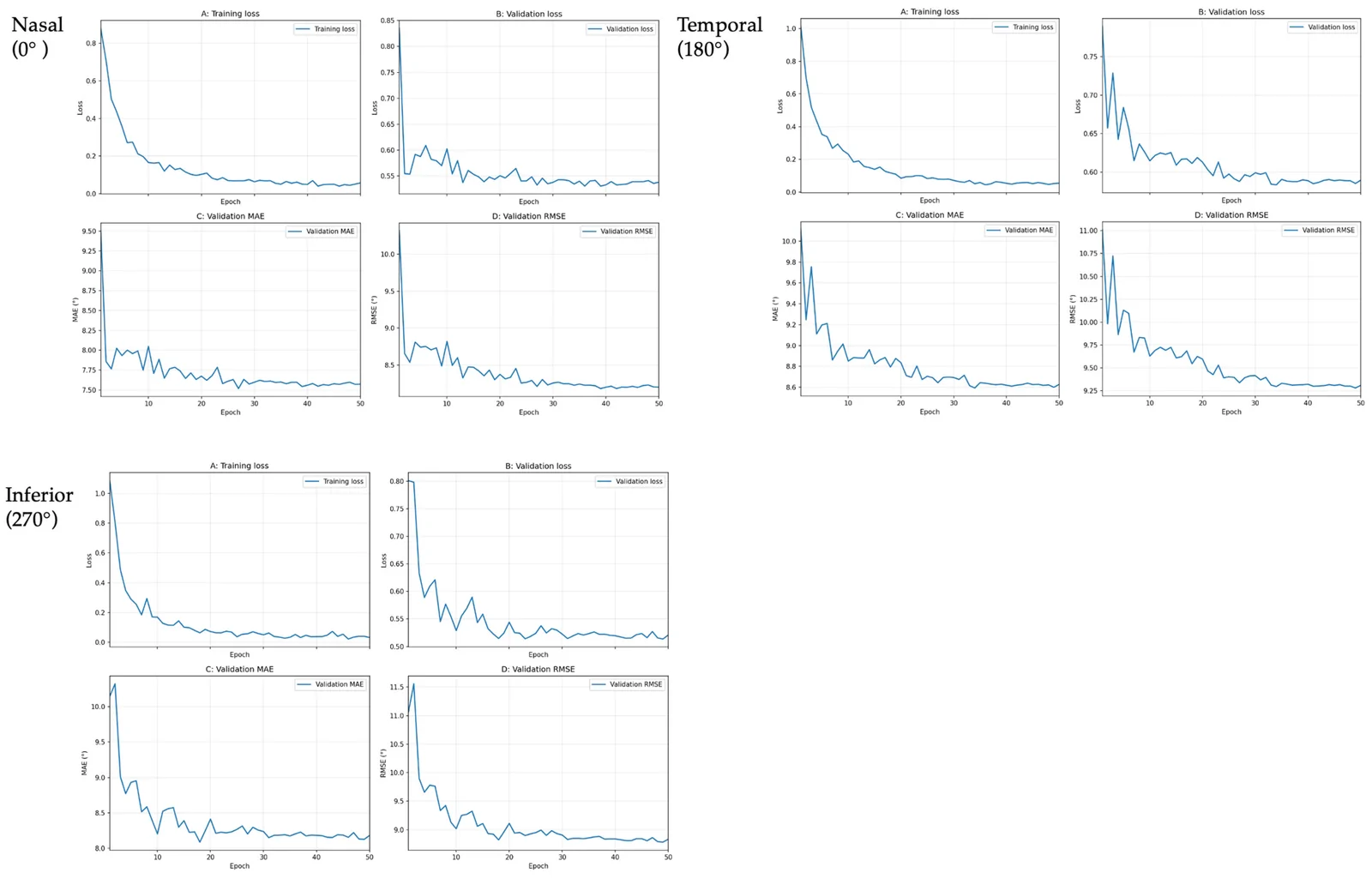
Training and validation learning curves for TIA500 estimation. Results are presented for the independently trained nasal (0°), temporal (180°), and inferior (270°) models. For each direction, panels show (**A**) training loss, (**B**) validation loss, (**C**) validation mean absolute error (MAE), and (**D**) validation root mean squared error (RMSE).

**Figure 4:**
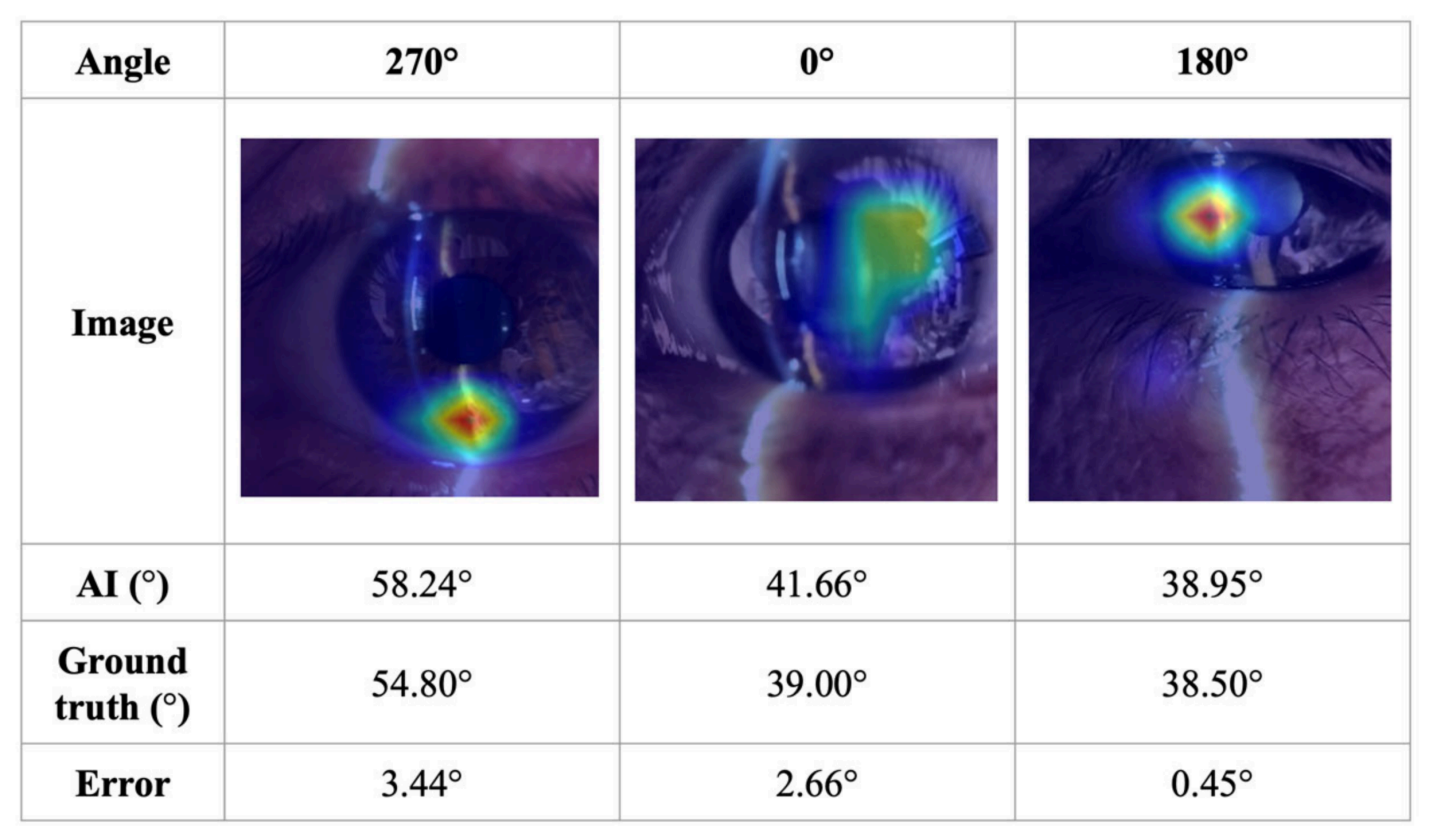
Representative AI-based trabecular–iris angle predictions and Grad-CAM visualizations. Representative smartphone-captured slit-lamp images obtained at 270° (inferior), 0° (nasal), and 180° (temporal) are overlaid with gradient-weighted class activation mapping (Grad-CAM) heatmaps. Warmer colors indicate image regions that contributed more strongly to the model’s predictions. AI-predicted TIA500 values, anterior-segment optical coherence tomography–derived ground-truth values, and absolute prediction errors are shown for each image.

## Data Availability

The data presented in this study are not publicly available due to privacy and ethical restrictions. Data may be made available from the corresponding author upon reasonable request.

## Abbreviations

The following abbreviations are used in this manuscript:

ACD	Anterior chamber depth
AI	Artificial intelligence
AS-OCT	Anterior-segment optical coherence tomography
Grad-CAM	Gradient-weighted class activation mapping
ICC	Intraclass correlation coefficient
MAE	Mean absolute error
PAC	Primary angle closure
PACD	Primary angle-closure disease
PACG	Primary angle-closure glaucoma
PACS	Primary angle-closure suspect
rMSE	Root mean squared error
SD	Standard deviation
SEC	Smart eye camera
TIA	Trabecular–iris angle
